# SOCIAL COHESION, TRANSPORTATION, AND PARTICIPATION IN SOCIAL ACTIVITIES AMONG OLDER ADULTS

**DOI:** 10.1093/geroni/igz038.788

**Published:** 2019-11-08

**Authors:** Kenzie Latham-Mintus, Keith Miller

**Affiliations:** 1 IUPUI, Indianapolis, Indiana, United States; 2 Indiana University, Indianapolis, Indiana, United States

## Abstract

Objectives: The purpose of this research is to examine the role that social cohesion and access (conceptualized as mobility and transportation) plays on participation in social activities (i.e., visiting friends/family, attending religious services, participating in organizations, and going out for enjoyment). Participation in valued, social activities promotes of well-being through social interactions and the maintenance of personally meaningful relationships and lifestyles. Methods: Data from the National Health and Aging Trends (NHATS) study were used. The NHATS is representative of U.S. Medicare recipients ages 65 and older. The NHATS collects information on health and participation as well as detailed environmental measures, which makes it well suited for this research. Results: Higher ratings of social cohesion were associated with higher cumulative odds of participating in social activities among older adults, net of sociodemographic characteristics, personal network size, neighborhood disorder, and health factors. Taking public transportation services and walking places were associated with higher cumulative odds of participating in social activities. An interaction between social cohesion and walking places was significant (p=0.002). Older adults who reported high levels of social cohesion and walked to get around their community were more likely to participate in social activities compared with those reporting low social cohesion and walking as a transportation. Discussion: This research provides evidence that socially cohesive neighborhoods enable greater access to social activities through transportation services. Offering a range of transportation services is only piece of creating an age-friendly community—older adults must also feel comfortable using these options.

